# HLA-E/*Mtb* specific CD4^+^ and CD8^+^ T cells have a memory phenotype in individuals with TB infection

**DOI:** 10.3389/fimmu.2024.1505329

**Published:** 2024-12-23

**Authors:** Linda Voogd, Catherine Riou, Thomas J. Scriba, Marjolein van Wolfswinkel, Krista E. van Meijgaarden, Kees L. M. C. Franken, Robert J. Wilkinson, Tom H. M. Ottenhoff, Simone A. Joosten

**Affiliations:** ^1^ Leiden University Center for Infectious Diseases, Leiden University Medical Center, Leiden, Netherlands; ^2^ Division of Medical Virology, Department of Pathology, Institute of Infectious Disease and Molecular Medicine, University of Cape Town, Cape Town, South Africa; ^3^ Centre for Infectious Diseases Research in Africa, Institute of Infectious Disease and Molecular Medicine and Department of Medicine, University of Cape Town, Cape Town, South Africa; ^4^ South African Tuberculosis Vaccine Initiative, Institute of Infectious Disease and Molecular Medicine, Division of Immunology, Department of Pathology, University of Cape Town, Cape Town, South Africa; ^5^ Tuberculosis Laboratory, The Francis Crick Institute, London, United Kingdom; ^6^ Department of Infectious Diseases, Imperial College London, London, United Kingdom

**Keywords:** Tuberculosis, HLA-E, T cells, immunophenotyping, spectral flow cytometry

## Abstract

**Introduction:**

Tuberculosis (TB) is the deadliest infectious disease worldwide and novel vaccines are urgently needed. HLA-E is a virtually monomorphic antigen presentation molecule and is not downregulated upon HIV co-infection. HLA-E restricted *Mtb* specific CD8^+^ T cells are present in the circulation of individuals with active TB (aTB) and *Mtb* infection (TBI) with or without HIV co-infection, making HLA-E restricted T cells interesting vaccination targets for TB.

**Methods:**

Here, we performed in-depth phenotyping of HLA-E/*Mtb* specific and total T cell populations in individuals with TBI and in individuals with aTB or TBI and HIV using HLA-E/*Mtb* tetramers.

**Results and Discussion:**

We show that HIV co-infection is the main driver in changing the memory distribution of HLA-E/*Mtb* specific CD4^+^ and CD8^+^ T cell subsets. HLA-E/*Mtb* specific CD4^+^ and CD8^+^ T cells were found to circulate with comparable frequencies in all individuals and displayed expression of KLRG1, PD-1 and 2B4 similar to that of total T cells. The presence of HLA-E/*Mtb* specific T cells in individuals with aTB and TBI highlights the potential of HLA-E as a vaccine target for TB.

## Introduction

Tuberculosis (TB), caused by infection with *Mycobacterium tuberculosis* (*Mtb*), is currently the deadliest infectious disease caused by a single pathogen (1). Vaccination with Bacillus Calmette-Guérin (BCG), the only licensed vaccine available, developed in 1921, fails to curb the global pandemic. Novel vaccines to protect against TB are therefore urgently needed. Protein subunit-based vaccination approaches against TB aim to induce antigen specific classical HLA-I and HLA-II restricted CD8^+^ and CD4^+^ T cell responses. However, the large diversity of HLA-I and II molecules between individuals complicates the selection of *Mtb* peptides that can be presented irrespective of the genetic background. In contrast, non-classical HLA-I molecules have limited allelic diversity, are recognized by donor unrestricted T cells (DURT) and are interesting vaccine targets to induce population-wide T cell responses that are necessary for protection (2). HLA-E is such a non-classical antigen presentation molecule with only two functional alleles expressed in humans, HLA-E*01:01 and *01:03. They differ by one amino acid located outside the peptide binding groove at position 107, suggesting that both alleles have a comparable peptide binding repertoire (3, 4). HLA-E presents peptides derived from leader sequences of classical HLA-I molecules and is a well-described ligand for the CD94/NKG2A and -C co-receptor complex expressed on natural killer (NK) cells. This way, NK cells scan for cells with dysregulated antigen presentation machineries such as tumor cells (5–7). In addition, HLA-E can present peptides with diverse residue compositions from pathogens and tumors to T cell receptors (TCR) (8–16). We showed recently that two HLA-E/*Mtb* peptides were recognized by multiple different TCR sequences within and between TB infected individuals. Moreover, HLA-E/*Mtb* restricted CD8^+^ T cells were detected in the circulation of individuals with *Mtb* infection (TBI) and active TB (aTB) with or without HIV and revealed an unorthodox phenotype reflected by the secretion of T-helper 2 associated cytokines (e.g., IL-4 and IL-13) (16). They could exert functional responses *in vitro*, such as controlling *Mtb* growth in infected macrophages (17). Memory phenotyping on HLA-E/*Mtb* CD8^+^ T cells previously revealed an effector memory and terminally differentiated phenotype, particularly in HIV/*Mtb* co-infected individuals (16). This induction of functional effector memory MHC-E CD8^+^ T cells was also observed in rhesus macaques that were vaccinated with a RhCMV-derived vector that encodes multiple *Mtb* antigens (18). Chronic antigen exposure may drive memory T cells into exhaustion, which is characterized by increased expression of exhaustion markers, such as KLRG-1, CTLA-4 and PD-1 (19). Signaling via these receptors impairs proliferation and functional capacities. In chronically *Mtb*-exposed individuals, such as in individuals with aTB and after BCG vaccination in healthy individuals, expression of exhaustion markers on CD4^+^ and CD8^+^ T cells has been reported, but whether this plays a role for HLA-E restricted T cells is currently unknown (20–22).

Recently, we identified several novel HLA-E/*Mtb* epitopes using an optimized peptide binding algorithm for HLA-E. These peptides, when bound to HLA-E, were recognized by a higher frequency of circulating CD8^+^ T cells in individuals with TBI compared to the previously identified and lower affinity HLA-E/*Mtb* epitopes (9). Moreover, we also demonstrated that not only CD8^+^ but surprisingly also CD4^+^ T cells could recognize these novel HLA-E/*Mtb* peptides, both in rhesus macaques (RM) and in humans, whereas control HLA-E/CMV complexes were only recognized by CD8^+^ T cells (23). In addition, we also showed that HLA-E/*Mtb* restricted CD4^+^ and CD8^+^ T cell frequencies remained unchanged in humans and RM after receiving BCG via intradermal or aerosol administration, similar to what was shown previously for other DURT subsets in BCG revaccinated adults (24). However, the frequency was significantly increased after *Mtb* challenge in unvaccinated RM (23). These novel findings suggest that HLA-E/*Mtb* restricted CD4^+^ and CD8^+^ T cells can be induced by *Mtb* infection but poorly upon BCG vaccination and encouraged us to elucidate the phenotype of HLA-E restricted T cells in humans with various stages of TB, including upon HIV co-infection.

The recent development of spectral flow cytometry has facilitated detailed characterization of immune phenotypes even when cell numbers are limited, as it enables detection of more than thirty markers simultaneously in single samples. We have recently optimized a high-dimensional flow cytometry panel to characterize the antigen specific immune phenotype using tetramers (TM) in combination with cell surface markers (25). Here, we applied this strategy to explore HLA-E/*Mtb* restricted T cell subsets in individuals with aTB or TBI and HIV, including in individuals with TBI, recognizing four newly identified HLA-E/*Mtb* epitopes (9). We show that, despite differences in cohorts, various CD4^+^ and CD8^+^ T cell memory subsets can recognize the HLA-E/*Mtb* epitopes. These memory HLA-E/*Mtb* CD4^+^ and CD8^+^ T cells expressed similar levels of markers associated with exhaustion as the total T cell population. Our study provides novel insights into the phenotype of HLA-E/*Mtb* specific T cell subsets circulating in individuals with various stages of TB infection and contributes to the exploration of HLA-E as a vaccine target.

## Materials and methods

### PBMC samples

Bio-banked peripheral blood mononuclear cell (PBMC) samples from individuals with TBI (n=40), individuals with TBI and HIV co-infection (TBI HIV^+^) (n=48) and individuals with active TB and HIV co-infection (aTB HIV^+^) (n=14) were used in the current study (**Supplementary Tables 1**-**3** for overview of study participants). Individuals with TBI, aged between 12 and 18 years, were part of the South African Adolescent Cohort Study (ACS) performed among high school students in Worcester, Western Cape Province, by the South African Tuberculosis Vaccine Initiative (SATVI, South Africa). In the ACS, TBI was measured by Quantiferon TB Gold in-tube assay. Adolescents were not tested for HIV due to ethical constraints, but the prevalence of HIV among adolescents with TB is extremely low and would be expected to be even lower in healthy young individuals, as was also reported previously (26). Although not tested, TBI adolescents were therefore considered HIV negative.

Individuals with TBI or aTB and HIV co-infection, aged between 21 and 63 years, were recruited at the Ubuntu Clinic, Site B, Khayelitsha (Cape Town, South Africa) between March 2017 and December 2018. Those in the aTB group all tested sputum Xpert MTB/RIF (Xpert, Cepheid, Sunnyvale, CA, USA) positive and had clinical symptoms and/or radiographic evidence of TB. All aTB individuals were drug sensitive and had received no more than one dose of anti-Tuberculosis treatment (ATT) at the time of baseline blood sampling. The TBI individuals were all asymptomatic, had a positive IFN-γ release assay (IGRA, QuantiFERON -TB Gold In-Tube, Qiagen, Hilden, Germany), tested sputum Xpert MTB/RIF negative and exhibited no clinical evidence of aTB. Sputum Xpert MTB/RIF, sputum culture for *Mtb*, CD4 count, HIV VL tests were performed by the South African National Health Laboratory Services. Treatment with anti-retroviral therapy (ART) was self-reported and is shown in **Supplementary Tables 2** and **3**. Because ART was self-reported and therefore likely not reliable, we considered individuals with less than 1000 HIV copies/mL as HIV suppressed and individuals with more than 1000 HIV copies/mL as HIV unsuppressed (**Supplementary Tables 2**, **3**).

Written informed consent was obtained from each adult individual. Written informed assent was provided by adolescent participants and written informed consent was provided by their parents or legal guardians. The study protocols in aTB and TBI individuals with HIV were approved by the University of Cape Town Human Research Ethics Committee (HREC 050/2015) and was conducted under DMID protocol no. 15-0047. The study protocols for the ACS were approved by the Human Research Ethics Committee of the University of Cape Town. PBMC samples were shipped in a liquid nitrogen dry shipper to the LUMC, The Netherlands for flow cytometry staining and acquisition.

### HLA-E/*Mtb* restricted peptide selection and HLA-E*01:03 tetramer folding

*Mtb*-derived HLA-E (MTBHLAE) restricted peptides were identified previously using an optimized predictive peptide binding algorithm for HLA-E and were folded into HLA-E*01:03 monomers for tetramerization (9). Correct folding of the monomers was confirmed with HPLC and staining on LILRB1 expressing cells, as described previously (27). Twelve MTBHLAE peptides were evaluated for CD3^+^ T cell recognition in individuals with TBI or TBI and HIV to select four peptides with the highest recognition (**Supplementary Table 4**). These twelve peptides have also been evaluated before in individuals with TBI (9). Similar to that study, the four peptides with the highest recognition were MTBHLAE_31 (VLPAKLILM), MTBHLAE_93 (RLEAVVMLL), MTBHLAE_34 (LLPIKIPLI) and MTBHLAE_63 (ILAFEAPEL) and were therefore selected for the phenotyping panels (**Supplementary Figure 1**). The four HLA-E*01:03 TM were divided into two separate staining pools as evaluation of single TM was not possible due to limited material availability. Pool 1 consisted of MTBHLAE_31 and MTBHLAE_93 and pool 2 consisted of MTBHLAE_34 and MTBHLAE_63. Staining with the high binding affinity HLA-E *Mtb*-restricted peptide 44 (RLPAKAPLL) was included as negative control as this peptide is poorly recognized in human PBMC. The HLA-E binding peptide from CMV (VLAPRTLLL) was included as comparator.

### Flow cytometry staining and acquisition

For the peptide selection, approximately 0.5 x 10^6^ PBMC per individual with TBI or TBI and HIV were stained with nine cell surface markers in combination with one of the twelve HLA-E/*Mtb* TM shown in **Supplementary Table 4** and samples were acquired on a BD LSRFortessa (BD Biosciences).

For in depth immune phenotyping analysis, a 30-colour panel was used for PBMC of individuals with: TBI (n=20), TBI and HIV (n=17) and aTB and HIV (n=14) (**Supplementary Table 5**). Due to availability problems with Pacific Orange CD20 after staining 12 individuals (5 individuals with TBI, 4 individuals with TBI and HIV and 3 individuals with aTB and HIV), this marker was changed to BV570 for the remaining 39 individuals. Only the samples stained with anti-CD20 BV570 were included in the total and HLA-E/*Mtb* specific immune subset analysis as combining samples stained with anti-CD20 BV570 or Pacific Orange would introduce significant compensation problems. To characterize the exhaustion profile of HLA-E/*Mtb* TM^+^ cells, a 20-colour panel was developed for PBMC of individuals with TBI (n=20) and TBI and HIV (n=31) (**Supplementary Table 6**). In both panels, 2 x 10^6^ PBMC per individual were used for staining with the two HLA-E/*Mtb* TM pools and 0.5x10^6^ PBMC per individual for staining with the two control HLA-E TM (p44; RLPAKAPLL and pCMV; VLAPRTLLL). Samples were acquired on a 5 laser Cytek^®^Aurora (Cytek Biosciences).

For all measurements, PBMC were first stained with LIVE/DEAD™ Fixable Violet or Blue (Invitrogen), depending on the panel, according to manufacturer’s instructions and blocked with 10 μg/mL purified CD94 monoclonal antibody (Clone HP-3D9, BD Biosciences) in phosphate buffered saline (PBS; Fresenius Kabi Nederland B.V) for 30 min at room temperature (RT) in the dark. PBMC were then washed (5 min, 450 x g) with PBS containing 0.1% bovine serum albumin (BSA; Roche Diagnostics GmbH) (PBS/0.1% BSA), blocked with 5% pooled normal human serum (Sigma-Aldrich) in PBS for 10 min at RT to block Fc receptors, preventing non-specific binding, and washed once with PBS/0.1% BSA. PBMC were subsequently incubated with a cocktail containing True-Stain Monocyte Blocker™ (1:20, BioLegend) to block non-specific binding of tandem-dyes, Brilliant Stain Buffer Plus (1:10, BD Biosciences) and chemokine receptor antibodies in PBS/0.1% BSA for 30 min at 37°C in the dark. After incubation, PBMC were washed once with PBS/0.1% BSA. For all samples, PBMC were incubated with HLA-E TM (5.4 µg/ml per TM) in PBS/0.1% BSA for 30 min at 37°C in the dark. PBMC were then washed, fixated with 1% paraformaldehyde (Pharmacy LUMC) for 10 min at RT and washed once more with PBS/0.1% BSA. PBMC were stained with a cocktail containing Brilliant Stain Buffer Plus (1:10) and the surface antibodies shown in **Supplementary Tables 4**-**6** in PBS/0.1% BSA for 30 min at 4°C in the dark. After incubation, PBMC were washed twice with PBS/0.1% BSA, fixated with 1% paraformaldehyde for 10 min at RT, washed once and resuspended in PBS/0.1% BSA before acquiring. Full gating strategies to determine HLA-E T cell frequencies and the immune phenotype are shown in **Supplementary Figures 2** and **5**.

### Data analysis of flow cytometry data

Flow cytometry data was analyzed using FlowJo v10.8.0 and OMIQ analysis software (www.omiq.ai). With OMIQ, data was first manually gated to remove debris, aggregates and dead cells. On the live single cells, a UMAP following FlowSOM clustering was performed to visualize and cluster the data. In the total immune profile analysis, subsampling was performed on 35087 events per sample (this was the lowest number of maximal events in one of the samples), and clusters with >100 events per sample were included for visualization. In the HLA-E/*Mtb* specific immune profile analysis, subsampling was done on a total of 5000 HLA-E TM^+^ events/cells per TB disease group (with a range of 55 to 285 events per sample). All markers except for LIVE/DEAD™, HLA-E TM, CD14, CD19 and CD20 were used in the UMAP settings and the resulting T cell parameters were included to cluster the data by FlowSOM. Only HLA-E TM^+^ T cells with more than 44 events were evaluated for exhaustion marker expression. This cut-off was based on the mean HLA-E CD3^+^ p44 counts plus twice the standard deviation and was used to exclude data skewing by including samples with too few TM counts.

### Statistical analysis

The statistical tests used were the Friedman Test, Multiple Mann-Whitney test, Spearman’s rank correlation and EdgeR. Statistical analysis was performed in GraphPad Prism v10.2.3 and OMIQ analysis software. The Figure legends describe the specific test used for each comparison.

## Results

### HIV co-infection changes the distribution of CD4^+^ and CD8^+^ T cells in individuals with TB infection

**Figure 1:**
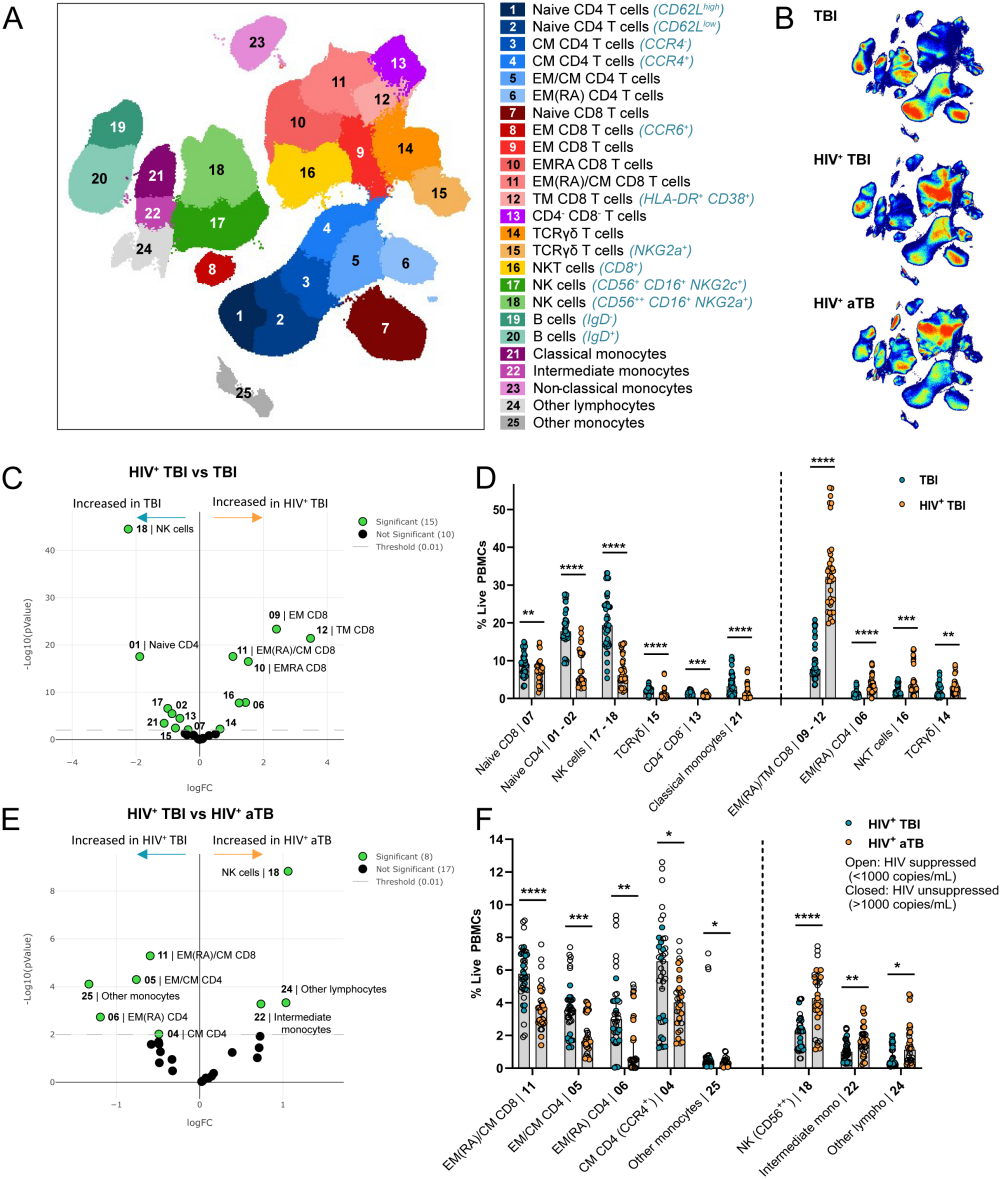
Individuals with TBI and HIV (n=13), aTB and HIV (n=11) or TBI (n=15) have differently distributed immune profiles. **(A)** UMAP visualization of 25 immune subsets identified using FlowSOM clustering on the total live PBMC population in all individuals combined. **(B)** UMAP density plots in individuals with TBI (top), TBI and HIV (middle) and aTB and HIV (bottom). **(C)** Volcano plot showing subsets that are significantly different between individuals with TBI and individuals with TBI and HIV in green and non-significant subsets in black. Significance was determined with EdgeR (p<0.01). **(D)** Bar graphs showing the frequency of live PBMC (Y-axis) in the subsets that are significantly different (X-axis) between individuals with TBI (blue circles) or TBI and HIV (orange circles). Bars show the median frequency and error bars the 95% confidence interval in each subset. Significance was tested using Multiple Mann-Whitney tests with multiple comparison correction (*p < 0.05; **p < 0.01; ***p < 0.001; ****p < 0.0001). **(E)** Same as **(C)**, comparing individuals with TBI or aTB with HIV. **(F)** Same as **(D)**, comparing individuals with TBI and HIV (blue circles) and aTB and HIV (orange circles).

Twenty-five common and rare immune cell subsets were identified in the circulation of individuals with TBI and HIV, aTB and HIV or TBI using FlowSOM clustering on the total live PBMC population (**Figure 1A**). HIV incidence among TBI adolescents is in general low and we therefore considered these individuals as HIV negative (26). Subsets were manually annotated based on the marker expression in each cluster (**Supplementary Figure 3**). Density plots of the UMAPs revealed that individuals with HIV had fewer CD4^+^ T cells (blue clusters in **Figure 1A**) compared to CD8^+^ T cell subsets (red clusters in **Figure 1A**) relative to individuals with TBI. Albeit our cohorts were not from the same population, these differences are consistent with what can be expected based on the pathophysiology of HIV (**Figure 1B**). Individuals with TBI had significantly more natural killer (NK) cells, naïve CD4^+^ and CD8^+^ T cells, TCRγδ T cells expressing NKG2A, CD4^-^ CD8^-^ T cells and classical monocytes and less terminally differentiated (EMRA) CD4^+^ and CD8^+^ T cells, transitional memory (TM) CD8^+^ T cells, NKT cells and γδ T cells than individuals with TBI and concomitant HIV (**Figures 1C, D**). Comparison between the individuals with HIV showed that individuals with TBI had significantly more EMRA, effector memory (EM) and central memory (CM) CD8^+^ and CD4^+^ T cells and fewer CD56^high^ NK cells and intermediate monocytes than individuals with aTB (**Figures 1E, F**). Consequently, individuals with TBI and HIV, aTB and HIV or TBI have different immune profiles, with an altered distribution of CD4^+^ and CD8^+^ T cells when co-infected with HIV, although we cannot fully exclude that the different demographics between individuals with HIV and TBI might also contribute to this difference.

### HLA-E/*Mtb* restricted peptides are recognized by both CD4^+^ and CD8^+^ T cells

**Figure 2:**
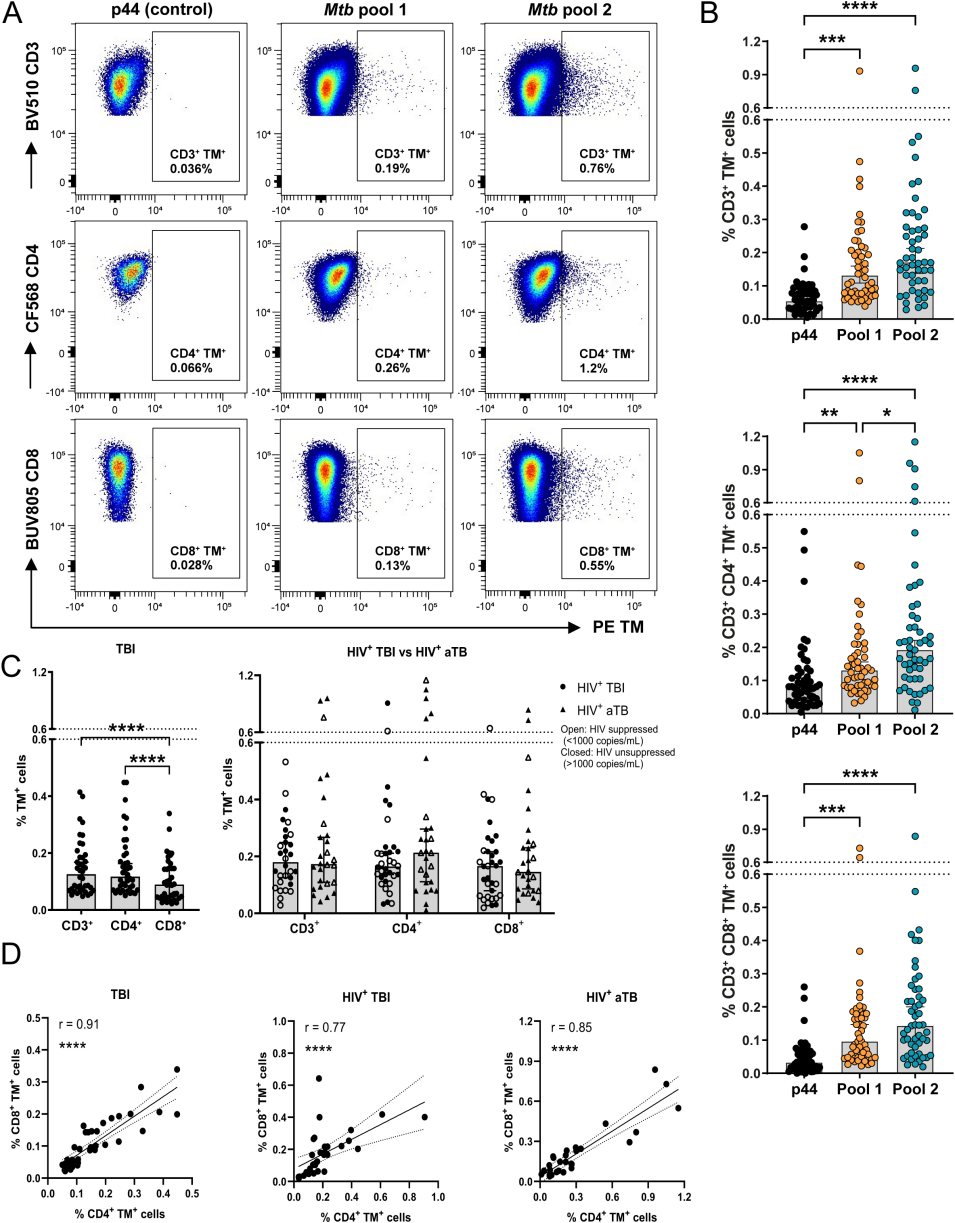
HLA-E/*Mtb* CD3^+^, CD4^+^ and CD8^+^ T cells are present in the circulation of individuals with TBI and HIV (n=17), aTB with HIV (n=14) or TBI (n=20). **(A)** Density plots for HLA-E p44, *Mtb* pool 1 and 2 CD3^+^, CD4^+^ and CD8^+^ T cells in one representative individual with aTB and HIV. HLA-E TM (PE) is shown on the X-axis and CD3, CD4 and CD8 on the Y-axis **(B)** Frequency of HLA-E p44, *Mtb* pool 1 and *Mtb* pool 2 CD3^+^ (top), CD4^+^ (middle) and CD8^+^ (bottom) T cells in the circulation of all individuals combined. Bars represent the median frequency and error bars the 95% confidence interval. Significance was tested using the Friedman test with multiple comparison correction (*p < 0.05; **p < 0.01; ***p < 0.001; ****p < 0.0001). **(C)** Frequency of HLA-E CD3^+^, CD4^+^ and CD8^+^ T cells recognizing *Mtb* pool 1 and 2 in individuals with TBI (left) and in individuals with TBI or aTB and HIV (right). Bars represent the median frequency and error bars the 95% confidence interval. Significance was tested using the Friedman test (left) and Multiple Mann-Whitney test (right) with multiple comparison correction (****p <0.0001). **(D)** Correlation plots showing the frequency of HLA-E CD4^+^ T cells on the X-axis and CD8^+^ T cells on the Y-axis recognizing *Mtb* pool 1 and 2 in individuals with TBI (left), TBI and HIV (middle) or aTB and HIV (right). Significance was tested with Spearman’s rank correlation (****p < 0.0001).

Twelve HLA-E/*Mtb* restricted peptides were evaluated for CD3^+^, CD4^+^ and CD8^+^ T cell binding in individuals with TBI and HIV or TBI. Four HLA-E restricted *Mtb*-derived peptides with the highest frequency of T cells in all individuals were selected for in-depth phenotypic analysis in individuals with TBI and HIV, aTB and HIV or TBI (**Supplementary Figure 1A**). These four peptides also displayed the highest recognition (among the similar set of peptides) in individuals with TBI previously, showing consistency (9). The HLA-E/*Mtb* peptides were divided into two pools while the high affinity binding HLA-E restricted *Mtb*-derived peptide 44 (p44) was separately included as a (negative) control. Representative density plots to determine HLA-E/*Mtb* restricted T cell frequencies for the *Mtb* pools and p44 are shown in **Figure 2A**. Both HLA-E/*Mtb* peptide pools were recognized at a significantly higher frequency of CD3^+^, CD4^+^ and CD8^+^ T cells compared to p44 (median T cell frequency circa 0.03% for p44 and more than 0.1% for both *Mtb* pools) (**Figure 2B**). Pool 2 was recognized at a significantly higher frequency of CD4^+^ T cells compared to pool 1, whereas pool 1 and 2 were recognized at a comparable frequency of CD3^+^ and CD8^+^ T cells (**Figure 2B**). CD4^+^ T cell recognition of HLA-E/*Mtb* peptides was further significantly higher than for CD8^+^ T cells within individuals with TBI (**Figure 2C**). T cell recognition of the HLA-E/*Mtb* peptides in general was comparable between all cohorts, despite different demographics between cohorts (**Figure 2C**). Recognition by CD4^+^ and CD8^+^ T cells strongly correlated, suggesting that both CD4^+^ and CD8^+^ T cells can recognize HLA-E/*Mtb* peptides, similar to our previous findings in BCG exposed individuals and RM (**Figure 2D**) (23). Altogether, CD4^+^ and CD8^+^ T cells can recognize HLA-E/*Mtb* peptides with comparable frequencies in individuals with TBI and HIV, aTB and HIV or TBI.

### HLA-E/*Mtb* specific CD4^+^ and CD8^+^ T cells have a memory phenotype in individuals with TBI and HIV, aTB and HIV or TBI

**Figure 3:**
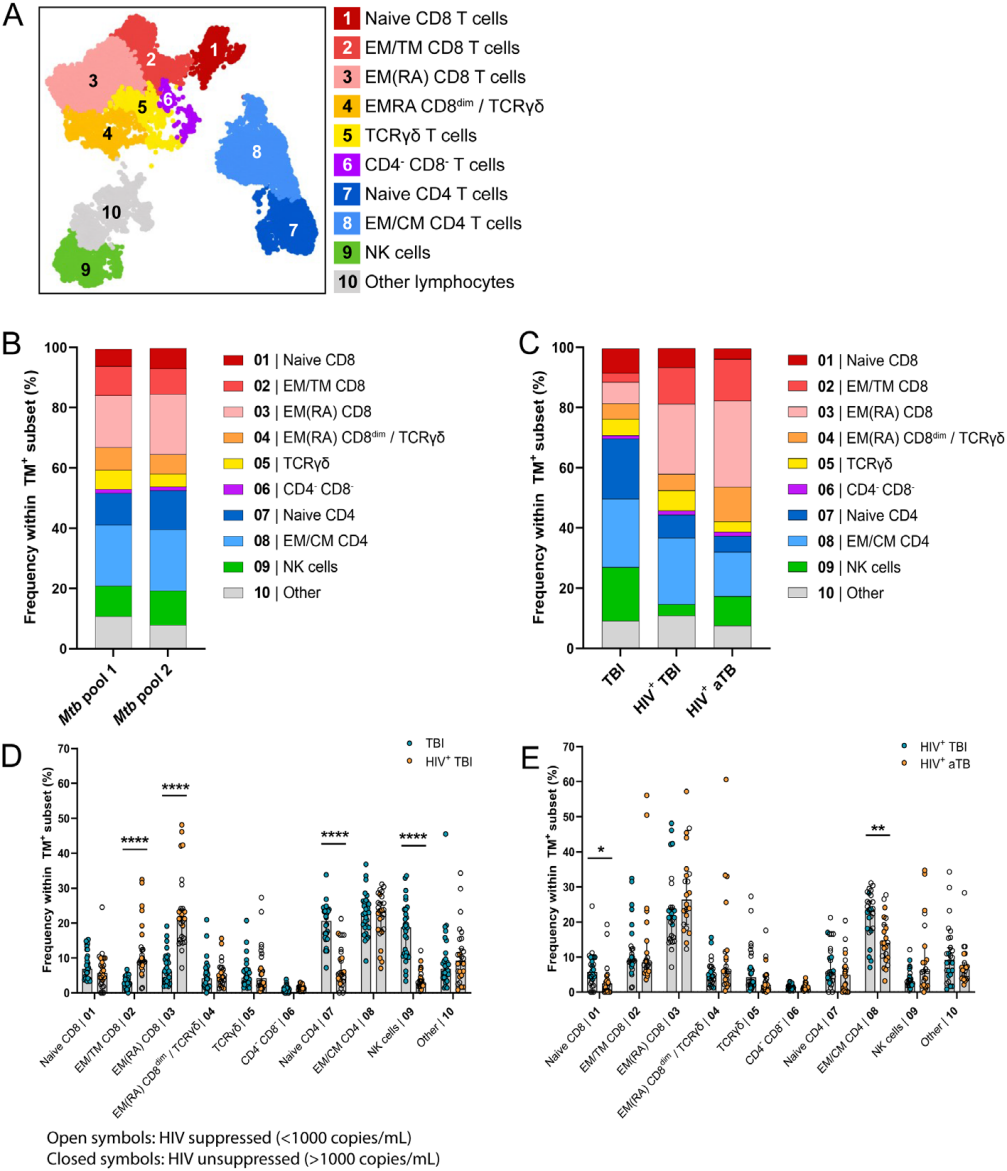
HLA-E/*Mtb* specific immune profile mainly consists of memory CD4^+^ and CD8^+^ T cells in individuals with TBI and HIV (n=13), aTB and HIV (n=11) or TBI (n=15). **(A)** UMAP visualization of 10 subsets identified using FlowSOM clustering on the total live HLA-E TM^+^ population recognizing *Mtb* pool 1 and pool 2 in all individuals combined. **(B)** Stacked bar graphs showing the mean frequency of HLA-E TM^+^ cells in each subset, separated for *Mtb* pool 1 and 2, all individuals combined (n=39). **(C)** Stacked bar graphs showing the mean frequency of HLA-E TM^+^ cells in each subset, separated per cohort. **(D)** Bar graphs showing the frequency of HLA-E TM^+^ cells recognizing *Mtb* pool 1 and 2 (Y-axis) in the 10 HLA-E/*Mtb* specific subsets (X-axis), separated for individuals with TBI (blue circles) or TBI and HIV (orange circles). Bars show the median frequency and error bars the 95% confidence interval. Significance was tested using Multiple Mann-Whitney tests with multiple comparison correction. *p < 0.05; **p < 0.01; ****p < 0.0001. **(E)** Same as **(D)**, comparing individuals with TBI and HIV (blue circles) and aTB and HIV (orange circles).

HLA-E/*Mtb* specific immune subsets were manually annotated based on the marker expression in each cluster (**Supplementary Figure 4A**). They comprised multiple CD4^+^ and CD8^+^ T cell memory subsets (i.e., EM, CM, Transitional Memory, EMRA and naïve T cells, possibly including stem cell memory T cells), γδ T cells, CD3^+^CD4^-^CD8^-^ T cells and NK cells irrespective of HIV or TB infection or disease (**Figure 3A**).

The distribution of HLA-E/*Mtb* specific immune subsets was similar for both HLA-E/*Mtb* peptide pools and events were therefore combined for further analysis (**Figure 3B**, **Supplementary Figure 4D**). The HLA-E/*Mtb* specific cell population predominantly consisted of CD4^+^ and CD8^+^ T cell memory subsets in all individuals with TBI or aTB (**Figure 3C**). HIV co-infection was the most dominant factor changing the distribution of CD4^+^ and CD8^+^ immune subsets as individuals with TBI and HIV had significantly more EM and EMRA CD8^+^ T cells and fewer naïve CD4^+^ T cells compared to individuals with TBI (**Figure 3D**, **Supplementary Figure 4B**), possibly due to HIV infection of CD4^+^ T cells, although individuals from these cohorts had different demographics. TB infection or disease had limited effects on the distribution of HLA-E/*Mtb* specific T cells, as the HLA-E/*Mtb* specific immune profile was almost identical between individuals with TBI or aTB with HIV (**Figure 3E**, **Supplementary Figure 4C**). Thus, despite the different demographics between cohorts, still very similar phenotypes were observed, describing HLA-E/*Mtb* T cells with a mixed memory phenotype.

### HLA-E/*Mtb* specific and total T cells have a comparable exhaustion marker expression in individuals with TBI and HIV or TBI

As HLA-E/*Mtb* restricted T cells have a memory phenotype, we evaluated the expression of the exhaustion markers KLRG1, PD-1 and 2B4 on an independent group of individuals with TBI or TBI and HIV. These markers are known to be expressed at the cell surface of memory T cells and provide information on the potential functionality of these memory HLA-E/*Mtb* T cells in individuals with TBI. Dual HLA-E TM staining was performed to enable optimal detection of low frequency circulating HLA-E restricted T cells for the HLA-E/*Mtb* peptides. The high affinity HLA-E restricted peptide from the viral UL40 protein of CMV (VLAPRTLL) was included as control for HLA-E/*Mtb* restricted T cell frequencies and exhaustion marker expression. Of note, the frequency of HLA-E T cells as determined by dual and single HLA-E TM staining was similar (**Supplementary Figure 5**, orange box).

**Figure 4:**
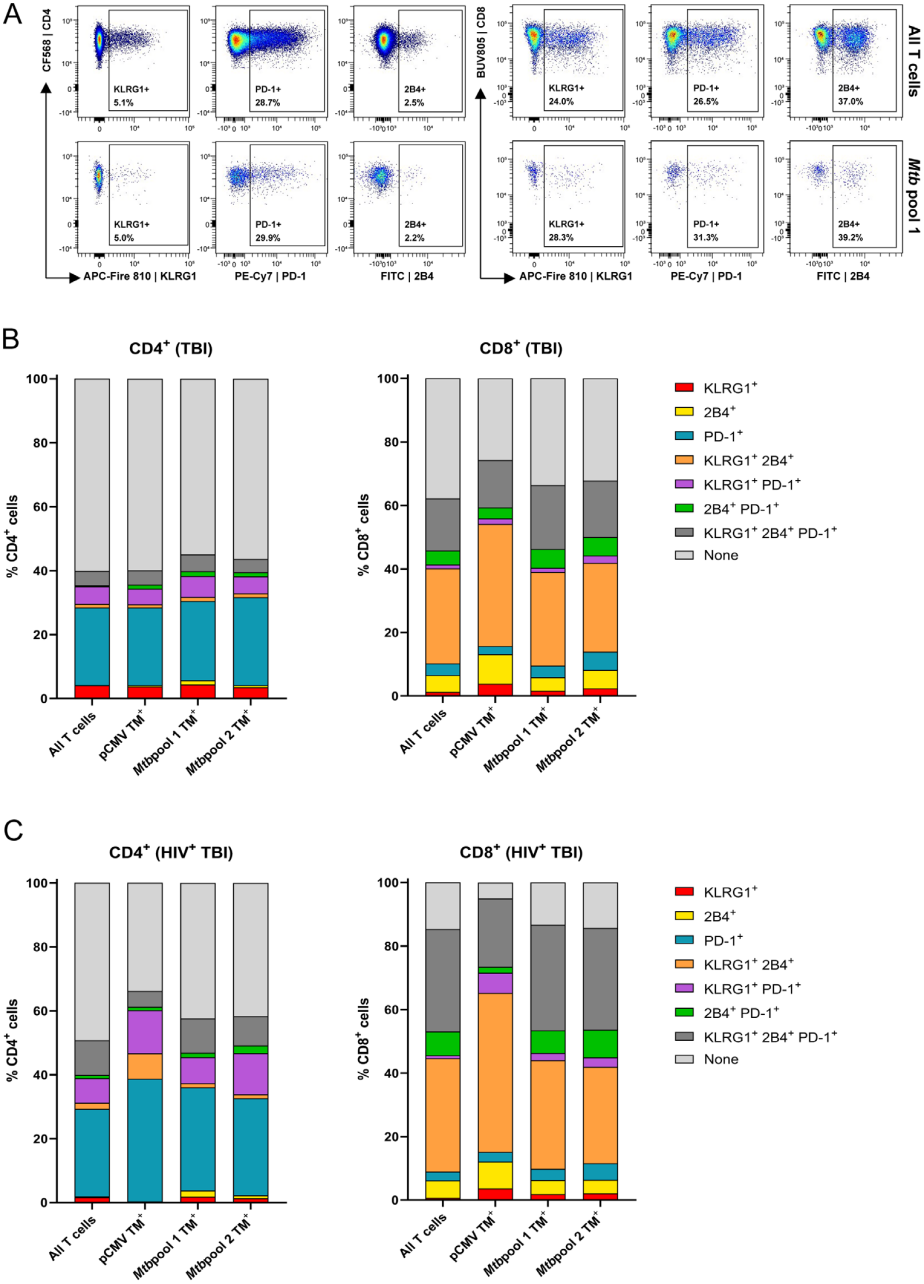
The exhaustion markers KLRG1, PD-1 and 2B4 are similarly expressed on HLA-E and the total T cells with differential expression between CD4^+^ and CD8^+^ T cells. **(A)** Density plots showing the expression of KLRG1, PD-1 and 2B4 on CD4^+^ (left) and CD8^+^ (right) T cells, comparing the total T cell population (top) and T cells recognizing HLA-E/*Mtb* pool 1 (bottom). Data is from one representative individual with TBI. **(B)** Stacked bar graphs showing the (co-)expression of KLRG1, 2B4 and PD-1 on HLA-E T cells recognizing pCMV (n=13), *Mtb* pool 1 (n=20) and 2 (n=19), compared to the total T cell population, in individuals with TBI. Stacks represent the mean expression frequency within CD4^+^ (left) and CD8^+^ (right) T cells. **(C)** Same as **(B)**, for individuals with TBI and HIV for *Mtb* pool 1 and 2 (n=27) and pCMV (n=7).

**Figure 4A** shows the expression of KLRG1, PD-1 and 2B4 on total and HLA-E/*Mtb* specific CD4^+^ and CD8^+^ T cells in one representative individual with TBI. Circa 40% of CD4^+^ T cells and 60% of CD8^+^ T cells expressed exhaustion markers with profound differences between these two subsets. Roughly 30% of CD4^+^ T cells expressed PD-1 as a single marker, whereas >30% of CD8^+^ T cells co-expressed KLRG1 and 2B4 and >10% co-expressed PD-1, KLRG1 and 2B4 simultaneously, both in individuals with TBI and HIV or TBI (**Figures 4B, C**). This co-expression of exhaustion markers was minimally observed on CD4^+^ T cells. The exhaustion marker signature was similar between total T cells, HLA-E/*Mtb* specific or HLA-E/CMV specific T cells. As expected, HLA-E/*Mtb* CD4^+^ and CD8^+^ T cells expressing exhaustion markers had a memory phenotype (**Supplementary Figure 6**). Altogether, HLA-E/*Mtb* T cells have a comparable exhaustion marker expression as the total T cell population.

## Discussion

We performed in-depth phenotyping on circulating HLA-E/*Mtb* specific and total T cells in individuals with various stages of TB infection and demonstrate that HIV co-infection is the main driver changing the distribution of CD4^+^ and CD8^+^ T cell memory subsets. In addition, we show that HLA-E restricted *Mtb* specific T cells have a mixed memory phenotype in all individuals.

HLA-E/*Mtb* specific cells are composed of memory CD4^+^ and CD8^+^ T cells, TCRγδ T cells and NK cells. Individuals with TBI and HIV had significantly more EM and EMRA CD8^+^ T cells and fewer NK cells compared to individuals with TBI, in both the HLA-E/*Mtb* restricted and total immune population. A lower frequency of NK cells and impaired functional NK cell responses was also observed previously in individuals with TBI and HIV compared to individuals with TBI only, pointing to a possible mechanism of HIV to prevent clearance of infected cells (28). The overall percentage of CD8^+^ T cells was higher in individuals with HIV and TB co-infection relative to individuals with TB infection in both total and HLA-E/*Mtb* restricted T cell subsets. This shift is most likely caused by HIV-mediated depletion of CD4^+^ T cells, which may skew the distribution towards more CD8^+^ T cells in individuals with HIV, even though individuals co-infected with HIV received anti-retroviral treatment and expression of HLA-E is not affected by HIV-infection (29). Expression of PD-1, KLRG1 and 2B4 was comparable between circulating HLA-E restricted T cells and total T cells in individuals with TBI or with TBI and HIV. These results suggest that HLA-E restricted T cells were not more exhausted than total T cells. Additional functional experiments, such as intracellular cytokine staining or proliferation assays, should be performed to substantiate the functionality of these HLA-E restricted T cells. In contrast to individuals with TBI and HIV or TBI, the HLA-E/*Mtb* specific immune profile revealed limited differences between individuals with TBI or aTB with HIV, suggesting that TB infection rather than disease has the strongest effect on the distribution of HLA-E/*Mtb* specific subsets.

The identification of NK cells in the HLA-E/*Mtb* restricted immune profile was most likely not mediated by CD94/NKG2A or -C as CD94 was blocked and expression of NKG2A and -C in the NK cell cluster was low. Although limited expression of TCRαβ in the NK cell subset, T cells that express NK cell markers could be partly responsible for identifying NK cells in the HLA-E/*Mtb* restricted immune profile. Alternatively, the interaction of NK cells with HLA-E/*Mtb* complexes might be mediated by other or unknown receptors. A possible candidate could be LILRB1, which is known to interact with the β2M subunit of HLA-E and is expressed on NK cells (30). TCRγδ T cells were also identified in the HLA-E/*Mtb* restricted immune profile. TCRγδ itself could be responsible for interaction with HLA-E/*Mtb* complexes as we showed previously that TCRγδ was expressed on 1 out of 13 HLA-E/*Mtb* restricted CD8^+^ T cell clones (17).

We observed differential expression of PD-1, KLRG1 and 2B4 on CD4^+^ and CD8^+^ T cells. The majority of CD4^+^ T cells expressed PD-1 as a single marker, whereas CD8^+^ T cells co-expressed exhaustion markers. Increased expression of PD-1 on CD4^+^ T cells relative to CD8^+^ T cells was also found previously in individuals with aTB (31). Several studies demonstrated that blockade of PD-1 increased the percentage of CD107a^+^ CD3^+^ T cells and the proliferation of CD4^+^ T cells in the circulation of individuals with aTB. PD-1 blockade also reduced BCG growth in infected monocyte derived macrophages from individuals with aTB (31, 32). However, treatment with PD-1 antagonists in TB infected individuals is under debate as a recent study in *Mtb*-infected RM demonstrated that anti-PD-1 treated RM had larger lung granulomas, increased bacterial loads and decreased CD4^+^ T cell functional responses in the granuloma compared to untreated RM (33). Also, it was reported in a meta-analysis of multiple studies that PD-1 and PD-L1 blockade treatment significantly increased the risk of TB reactivation in individuals with TBI (34). As targeting exhaustion markers in TB infected individuals can have severe consequences, it would be interesting to determine the effect on vaccine induced immunity in TB naïve individuals.

HLA-E/*Mtb* T cell frequencies identified by dual and single HLA-E TM staining were similar, which underscores that single HLA-E TM can be included in panels with restricted fluorochrome options. Dual HLA-E TM staining is however preferred as it facilitates detection of lower circulating HLA-E T cell frequencies and facilitates discrimination between TM positive and negative populations. Furthermore, we demonstrate that the frequency of HLA-E/*Mtb* CD4^+^ and CD8^+^ T cells was comparable between individuals with TBI, TBI and HIV or aTB and HIV. In contrast, we showed previously that Italian individuals with aTB and HIV had the highest frequencies of HLA-E/*Mtb* restricted CD8^+^ T cells compared to individuals with TBI without HIV (9, 16). This discrepancy may result from difference in group size (i.e., five Italian individuals with aTB and HIV compared to twenty South African individuals with aTB and HIV in the current study), environmental or geographical factors, levels of exposure to TB, prior BCG vaccination or genetic backgrounds.

The phenotype of HLA-E/*Mtb* specific T cells and frequencies were evaluated in the circulation, but we showed previously that HLA-E/*Mtb* T cell frequencies were higher in the bronchoalveolar lavage (BAL) fluid, thus the local infection site, compared to the circulation in RM and healthy humans receiving BCG and/or *Mtb* challenge (23). This increase might be due to migration of HLA-E/*Mtb* T cells to the primary infection site. Trafficking of CD4^+^ T cells to the lung parenchyma was also shown before in *Mtb*-infected mice (35, 36). It would be interesting to study if similar increases of HLA-E/*Mtb* T cells in the BAL occurs in TB infected individuals as well, although this might be ethically challenging, including a comparison of the HLA-E/*Mtb* specific immune profile between the local site and the circulation.

HLA-E restricted *Mtb* peptides are recognized by both CD4^+^ and CD8^+^ T cells, shown here and in our previous study (23). This dual recognition raises questions about the thymic selection of HLA-E T cells, which might depend on the peptide sequence as high-affinity leader sequence derived peptides and the identical peptide mimic from CMV are only recognized by CD8^+^ T cells. We encourage future work to investigate the selection process in more detail.

One of the limitations of our study is that the analyses were performed on samples from a single time point per individual and only from South African individuals. Samples from different cohorts were included to investigate and compare individuals with TBI and HIV co-infection to individuals with TBI. The order of infection with HIV and TB in these individuals is unknown as is information on *Mtb* and HIV exposure and bacterial/viral load. Moreover, individuals with TBI or with TBI and HIV were recruited at different sites within South Africa and had different ages, hampering direct comparison of HIV status, however, the phenotypes observed were very similar despite differences in the populations investigated. Moreover, the frequency of HLA-E/*Mtb* T cells for enumeration and validation of the HLA-E/*Mtb* restricted immune profiles was low, unsurprisingly, and could be increased by including more HLA-E/*Mtb* peptides. Besides this, we could not compare absolute HLA-E/*Mtb* T cell counts as we only stained PBMC samples and not whole blood samples. Another limitation is that additional exhaustion markers, such as LAG-3, TIM3, CD57 and TOX-1, could have been included for a more extensive analysis of the exhaustion marker profile. Finally, functional assessment of the HLA-E/*Mtb* restricted T cells was not possible due to limited material availability. However, we previously performed extensive characterization on the function of HLA-E/*Mtb* restricted CD8^+^ T cells in individuals with TB infection or aTB. These studies showed that HLA-E/*Mtb* restricted CD8^+^ T cells have an unorthodox phenotype relative to classical *Mtb* restricted T cells, characterized by the secretion of T-helper 2 cytokines (i.e., IL-4 and IL-13) and the exertion of cytolytic or suppressive responses (16, 17). Additionally, in another study we showed that sorted HLA-E/*Mtb* TM^+^ CD8^+^ T cells in individuals from the same TB infection cohort as investigated in this study have a diverse TCR repertoire with limited clonal expansions, unexpected for a DURT member (8). Transduction of these TCRs confirmed the functionality of the identified HLA-E/*Mtb* TM^+^ CD8^+^ TCRs. Thus, although not assessed in this study, HLA-E/*Mtb* restricted T cells derived from individuals with TB can exert functional responses.

Taken together, we show that HLA-E/*Mtb* specific CD8^+^ and CD4^+^ T cells have memory phenotypes and express similar levels of exhaustion markers as total T cells. We therefore highly encourage further exploration of HLA-E as a vaccination target against TB.

## Data Availability

The original contributions presented in the study are included in the article/**Supplementary Material**. FSC files are deposited in FlowRepository (flowrepository.org) with ID number: FR-FCM-Z8MN. Further inquiries can be directed to the corresponding author.
