# Supplementary material for: HLA-E/Mtb specific CD4+ and CD8+ T cells have a memory phenotype in individuals with TB infection
Source: Front Immunol. 2024 Dec 23;15:1505329. doi: 10.3389/fimmu.2024.1505329 (PMC11714851; doi:10.3389/fimmu.2024.1505329)
Supplement: Supplementary file 1 [file DataSheet1.docx]

# Supplementary material

**Supplementary Figures**

**
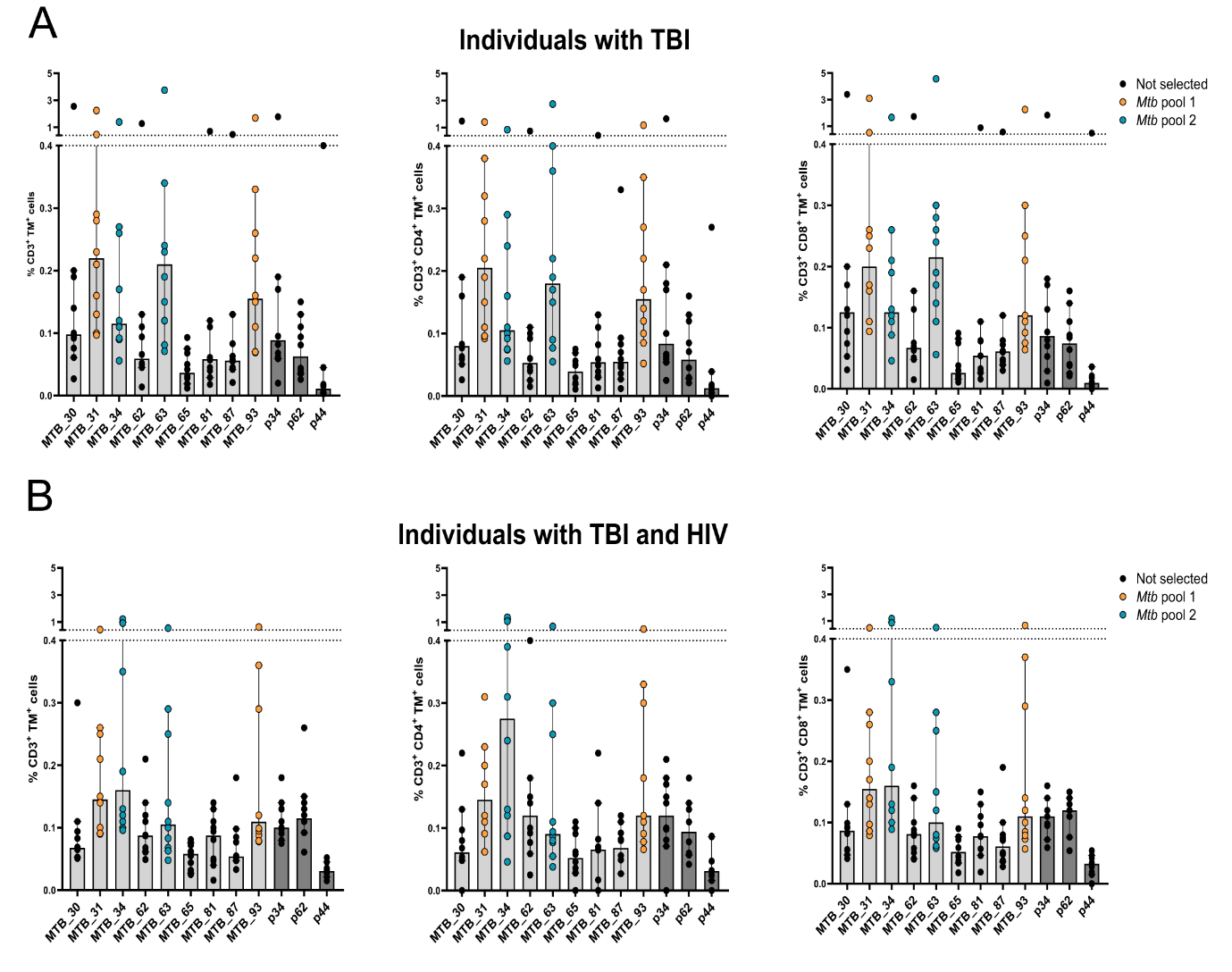
**

**Supplementary Figure 1: Selection of HLA-E/*Mtb* peptides based on CD3^+^, CD4^+^ and CD8^+^ T cell binding. A:** T cell binding in individuals with TBI (n=10)**. B.** T cell binding in individuals with TBI and HIV (n=10)**.** Orange dots represent the peptides selected for *Mtb* pool 1 and blue dots for *Mtb* pool 2. Black dots represent unselected peptides. Shaded bars represent the median frequency and error bars the 95% confidence interval.


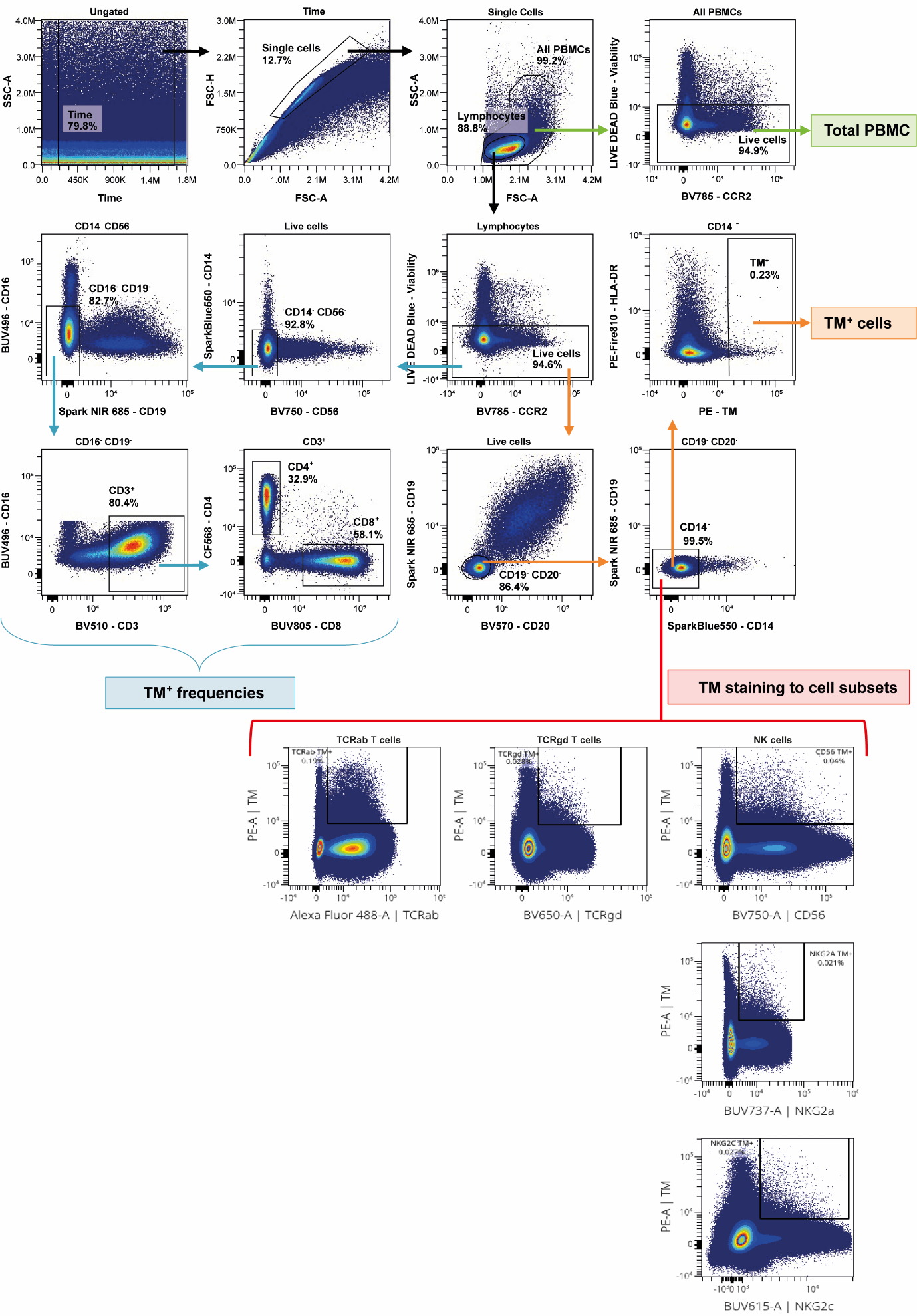


**Supplementary Figure 2: Gating strategy to determine the total live PBMC population (green), total HLA-E TM^+^ population (orange) and HLA-E CD3^+^, CD4^+^ and CD8^+^ T cell frequencies (blue) on one representative individual with aTB and HIV using the markers shown in Supplementary Table 4.** In addition, HLA-E TM staining on NK cells, TCRγδ T cells, and TCRαβ T cells as comparison, in the total live PBMC population is shown in red.


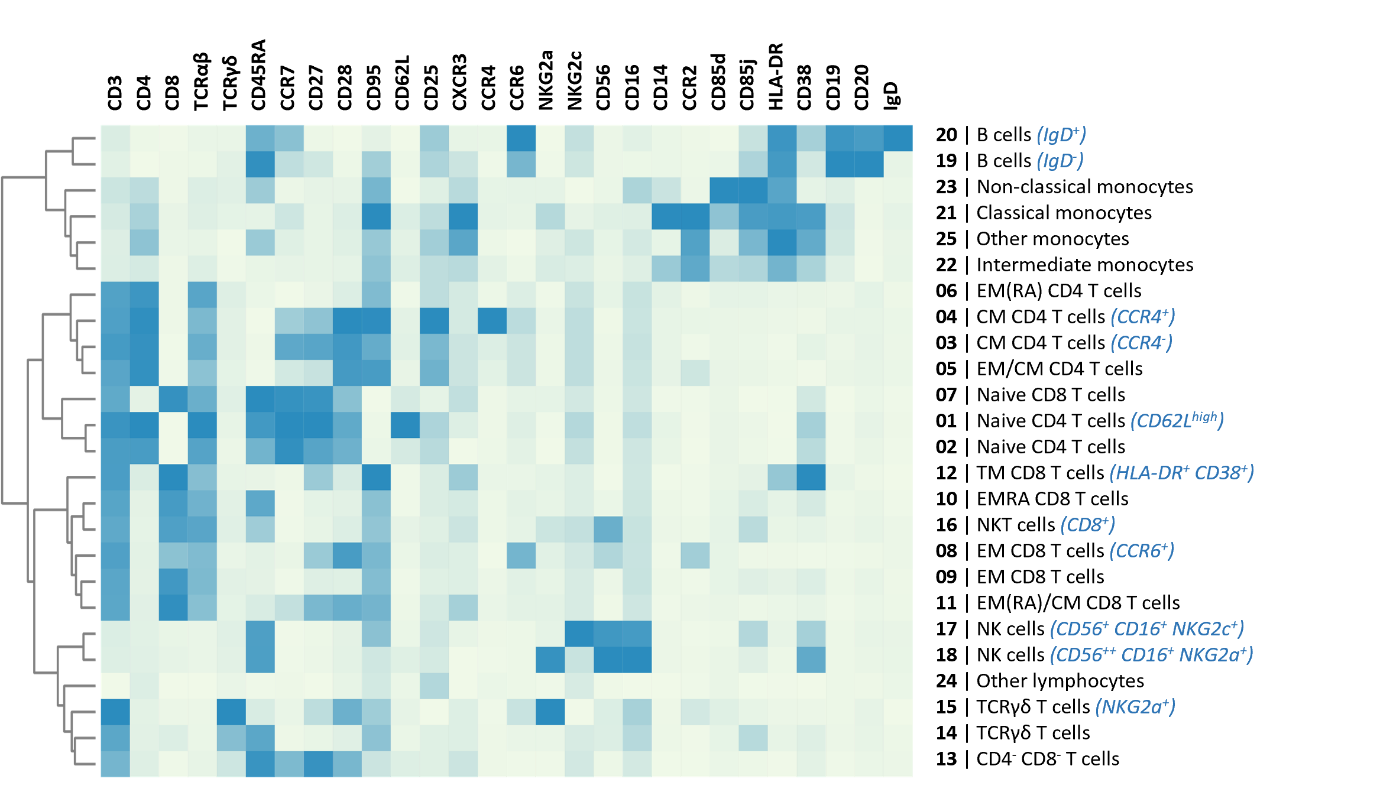
**Supplementary Figure 3: Expression of panel markers in the immune subsets present in the total immune profile.**


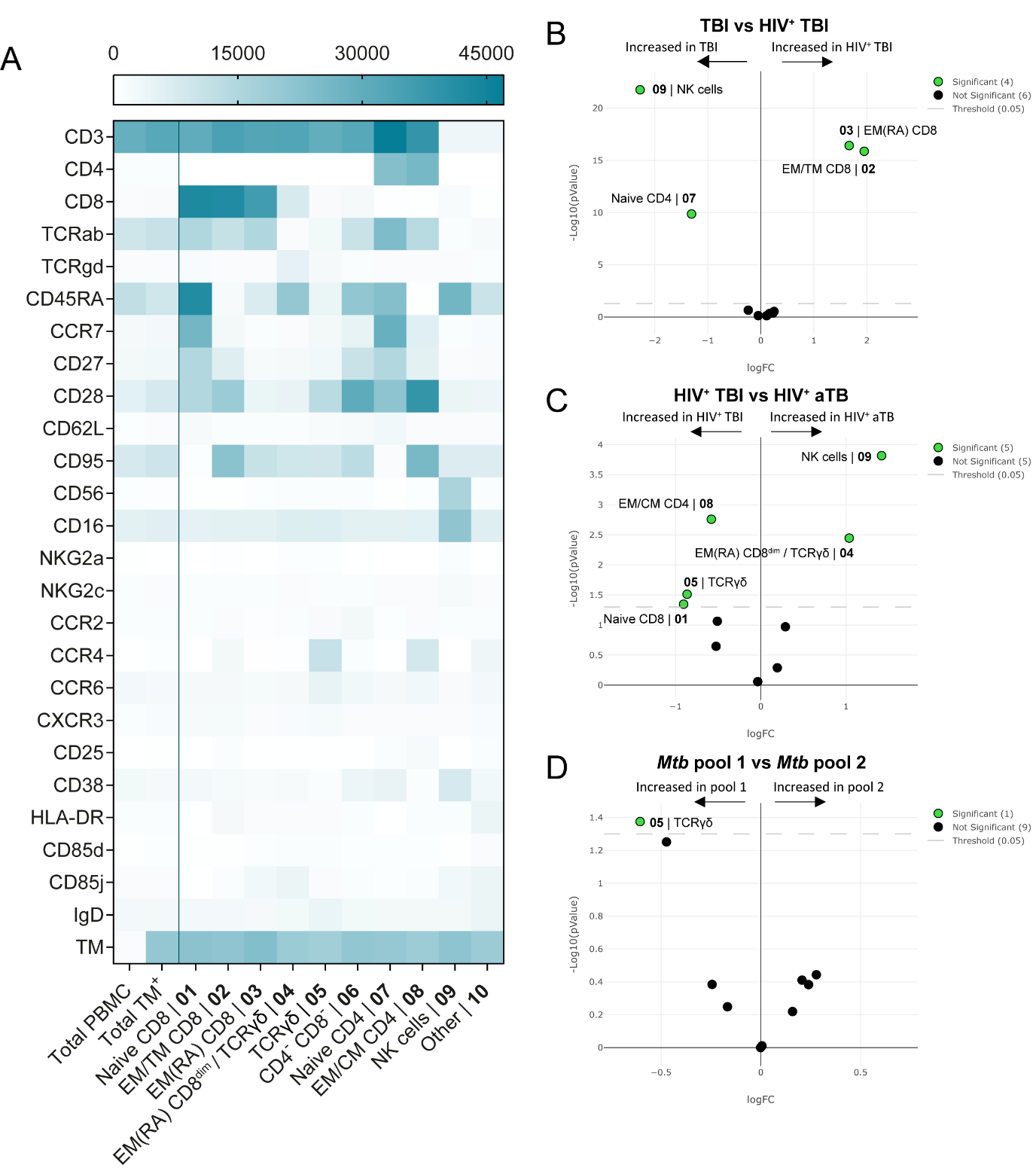


**Supplementary Figure 4. Expression of markers in HLA-E/*Mtb* specific immune subsets and comparison of the subsets between TB groups and *Mtb* TM pools. A:** Heatmap showing the median expression of each marker (Y-axis) in the 10 HLA-E/*Mtb* specific immune subsets (X-axis), all individuals combined. **B**: Volcano plot showing subsets that are significantly different between individuals with TBI and individuals with TBI and HIV in green and non-significant subsets in black. Significance was determined with EdgeR (p<0.05). **C**: Same as B, comparing individuals with TBI and HIV and individuals with aTB and HIV. **D**: Same as B, comparing *Mtb* pool 1 and 2, all individuals combined.


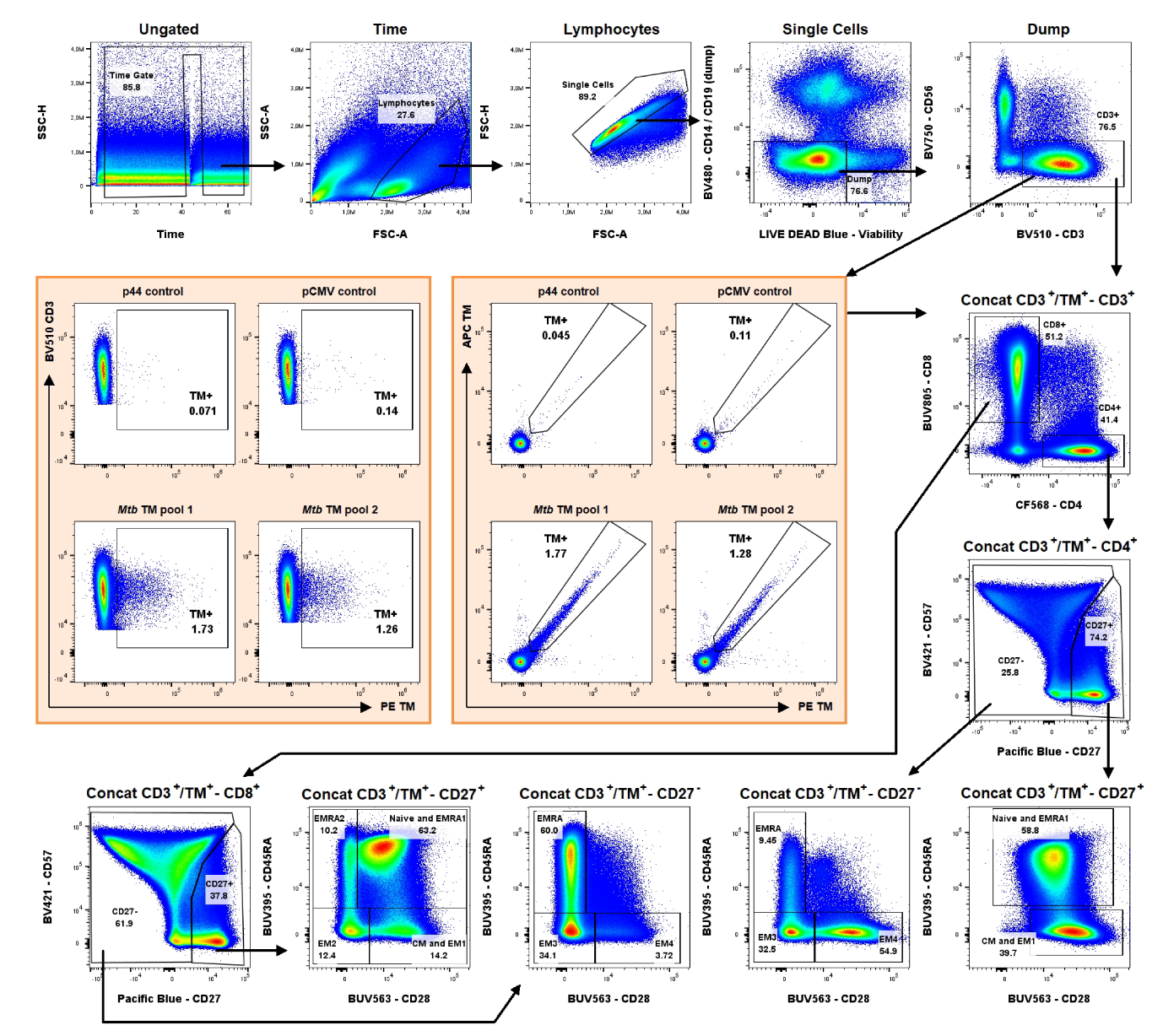


**Supplementary Figure 5. Gating strategy to determine HLA-E T cell frequencies using dual HLA-E TM staining and their memory phenotype.** Plots in orange show the comparison between the detection of HLA-E T cells based on dual TM staining or single staining. Data is from one representative individual with TBI.


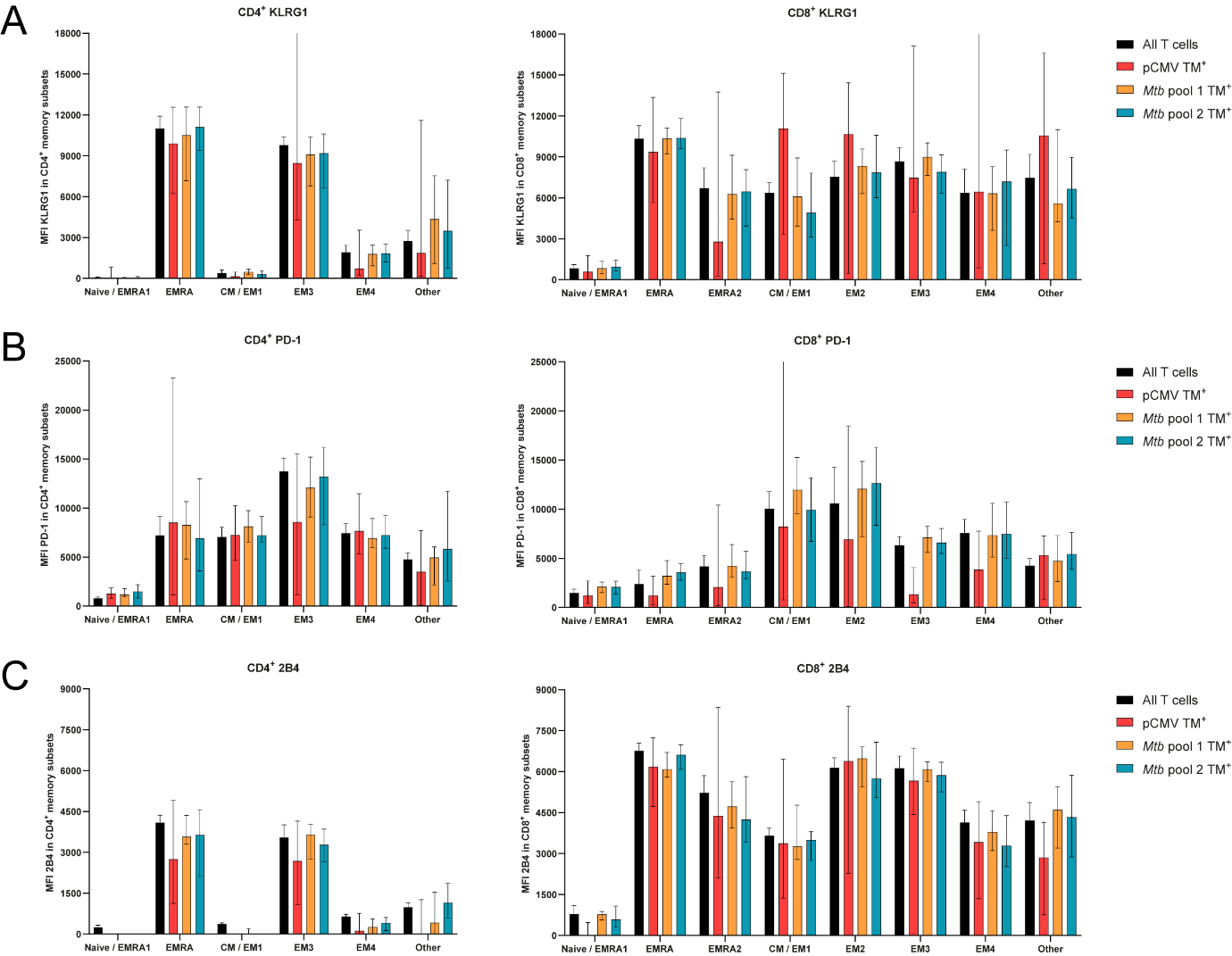


**Supplementary Figure 6. Mean fluorescent intensity (MFI) of KLRG1 (top), PD-1 (middle) and 2B4 (bottom) within the HLA-E CD4^+^ (left) and CD8^+^ (right) T cell memory phenotypes shown on the X-axis.** Bars represent the median MFI and the error bars the 95% confidence interval of the exhaustion marker expression based on the data in all individuals combined.

**Supplementary Tables**

**Supplementary Table 1. Overview of the individuals with TBI included in the immune repertoire or functional phenotyping analysis.** M=male; F=female.

| **TBI** | | | |
| --- | --- | --- | --- |
| **Participant no.** | **Age** | **Gender** | **Ethnicity** |
| **Immune repertoire analysis** | | | |
| 1 | 15 | F | coloured |
| 2 | 17 | F | coloured |
| 3 | 17 | F | coloured |
| 4 | 13 | F | black |
| 5 | 14 | F | black |
| 6 | 13 | F | black |
| 7 | 16 | M | coloured |
| 8 | 16 | F | coloured |
| 9 | 17 | F | black |
| 10 | 14 | F | coloured |
| 11 | 13 | F | coloured |
| 12 | 15 | M | coloured |
| 13 | 14 | M | coloured |
| 14 | 18 | F | coloured |
| 15 | 16 | F | coloured |
| 16 | 16 | F | coloured |
| 17 | 18 | F | coloured |
| 18 | 18 | M | coloured |
| 19 | 17 | M | coloured |
| 20 | 15 | F | coloured |
| **Functional phenotyping** | | | |
| 1 | 15 | M | coloured |
| 2 | 15 | M | black |
| 3 | 14 | F | coloured |
| 4 | 14 | F | coloured |
| 5 | 14 | F | coloured |
| 6 | 16 | M | coloured |
| 7 | 14 | F | coloured |
| 8 | 16 | M | coloured |
| 9 | 14 | M | coloured |
| 10 | 15 | M | coloured |
| 11 | 16 | M | coloured |
| 12 | 14 | M | coloured |
| 13 | 16 | F | coloured |
| 14 | 16 | M | coloured |
| 15 | 17 | F | coloured |
| 16 | 15 | M | white |
| 17 | 16 | M | coloured |
| 18 | 14 | F | coloured |
| 19 | 16 | M | coloured |
| 20 | 15 | M | coloured |

**Supplementary Table 2. Overview of the individuals with TBI and HIV included in the immune repertoire or functional phenotyping analysis.** ART=Anti-retroviral therapy; IGRA=Interferon Gamma Release Assay; VL=viral load (copies/mL); M=male; F=female; Y=yes; N=no. Treatment with ART was self reported.

| **HIV^+^ TBI** | | | | | | | | |
| --- | --- | --- | --- | --- | --- | --- | --- | --- |
| **Participant no.** | **Age** | **gender** | **ART (self reported)** | **IGRA** | **IGRA (IU/ml)** | **Gene Expert** | **CD4 count (10^6^/l)** | **HIV VL**  **(copies/mL)** |
| **Immune repertoire analysis** | | | | | | | | |
| 1 | 29 | F |  | POS | 28,4 | nd | 749 | 38185 |
| 2 | 43 | F |  | POS | 1,76 | NEG | 989 | 33054 |
| 3 | 22 | F |  | POS | 2,81 | NEG | 627 | 6865 |
| 4 | 45 | F |  | POS | 1,33 | NEG | 695 | 103804 |
| 5 | 51 | M | N | POS | >10 | NEG | 500 | 94153 |
| 6 | 42 | F | Y | POS | 8,4 | NEG | 655 | <20 |
| 7 | 32 | F | Y | POS | 2,05 | NEG | 454 | <20 |
| 8 | 33 | F | Y | POS | >10 | NEG | 478 | <20 |
| 9 | 63 | M | Y | POS | 9,67 | NEG | 417 | <20 |
| 10 | 28 | F | N | POS | 0,29 | NEG | 256 | 33657 |
| 11 | 37 | F | Y | POS | 7,62 | NEG | 412 | 98701 |
| 12 | 24 | F | Y | POS | 0,7 | NEG | 706 | <20 |
| 13 | 36 | F | N | POS | 0,43 | NEG | 283 | 10509 |
| 14 | 45 | F | Y | POS | 0,92 | NEG | 395 | <20 |
| 15 | 41 | F | Y | POS | 1,66 | NEG | 765 | <20 |
| 16 | 35 | F | Y | POS | 3,54 | NEG | 446 | <20 |
| 17 | 32 | F | Y | POS | 0,65 | NEG | 346 | 8830 |
| **Functional phenotyping** | | | | | | | | |
| 1 | 44 | F |  | POS | 1,89 | NEG | 387 | 26054 |
| 2 | 30 | F |  | POS | 1,06 | NEG | 577 | <20 |
| 3 | 32 | F |  | POS | 2,49 | NEG | 373 | 301 |
| 4 | 38 | F | Y | POS | 0,35 | NEG | 442 | 82312 |
| 5 | 30 | M | Y | POS | >10 | NEG | 563 | 113654 |
| 6 | 23 | F |  | POS | na | NEG | 327 | 160840 |
| 7 | 37 | F | Y | POS | >10 | NEG | 716 | 24 |
| 8 | 34 | F |  | POS | 3,33 | NEG | 649 | <20 |
| 9 | 26 | M |  | POS | na | NEG | 451 | 556154 |
| 10 | 45 | M |  | POS | 0,79 | NEG | 433 | 45232 |
| 11 | 29 | F |  | POS | 1,09 | NEG | 543 | 68195 |
| 12 | 50 | F |  | POS | 20,9 | NEG | 674 | 316 |
| 13 | 28 | F |  | POS | 12,1 | NEG | 626 | 4837 |
| 14 | 38 | F |  | POS | 5,8 | NEG | 1066 | 211 |
| 15 | 31 | F |  | POS | 3,5 | NEG | 464 | 491782 |
| 16 | 33 | F |  | POS | >10 | NEG | 551 | 11649 |
| 17 | 36 | F | Y | POS |  | NEG | 654 | 98 |
| 18 | 46 | M |  | POS | 1,34 | NEG | 563 | 820866 |
| 19 | 26 | M |  | POS | 12,97 | NEG | 406 | 1012 |
| 20 | 35 | F | Y | POS | 1,03 | NEG | 263 | 2617 |
| 21 | 44 | F | Y | POS | 0,77 | NEG | 121 | 1403 |
| 22 | 42 | M | Y | POS | 0,85 | NEG | 270 | <20 |
| 23 | 21 | F | Y | POS | 0,56 | NEG | 577 | <20 |
| 24 | 34 | F | Y | POS | 0,5 | NEG | 389 | <20 |
| 25 | 38 | M | Y | POS | 5,62 | NEG | 353 | 1805 |
| 26 | 42 | F | Y | POS | 0,45 | NEG | 616 | <20 |
| 27 | 33 | F | Y | POS | >10 | NEG | 275 | 2956 |
| 28 | 51 | F | N | POS | 5,46 | NEG | 969 | 24691 |
| 29 | 38 | F | Y | POS | >10 | NEG | 551 | <20 |
| 30 | 48 | F | Y | POS | 5,91 | NEG | 723 | <20 |
| 31 | 36 | F | Y | POS | 6,44 | NEG | 354 | 56 |

**Supplementary Table 3. Overview of the individuals with aTB and HIV included in the immune repertoire analysis.** ART=Anti-retroviral therapy; IGRA=Interferon Gamma Release Assay; VL=viral load (copies/mL); M=male; F=female; Y=yes; N=no. Treatment with ART was self reported.

| **HIV^+^ aTB** | | | | | | | | |
| --- | --- | --- | --- | --- | --- | --- | --- | --- |
| **Participant no.** | **Age** | **gender** | **ART (self reported)** | **IGRA** | **IGRA (IU/ml)** | **Gene Expert** | **CD4 count (10^6^/l)** | **HIV VL**  **(copies/mL)** |
| **Immune repertoire analysis** | | | | | | | | |
| 1 | 46 | M | N | na |  | POS | 205 | 3484818 |
| 2 | 24 | M | N | na |  | POS | 149 | 335470 |
| 3 | 50 | M | N | na |  | POS | 105 | 38752 |
| 4 | 36 | F | N | na |  | POS | 157 | 86034 |
| 5 | 33 | F | N | na |  | POS | 408 | 103011 |
| 6 | 21 | F | N | na |  | POS | 212 | 18313 |
| 7 | 55 | F |  | na |  | POS | 999 | 46 |
| 8 | 31 | F |  | na |  | POS | 206 | 49272 |
| 9 | 41 | F | Y | na |  | POS | 460 | 1000 |
| 10 | 49 | M | Y | na |  | POS | 374 | <20 |
| 11 | 58 | M | N | na |  | POS | 52 | 361424 |
| 12 | 24 | F | Y | na |  | POS | 374 | <20 |
| 13 | 45 | F | N | na |  | POS | 317 | 67947 |
| 14 | 47 | M | Y | na |  | POS | 261 | 284 |

**Supplementary Table 4. Flow cytometry panel to select *Mtb* peptides for inclusion in the in-depth immune phenotyping panels.**

| **Marker** | **Fluorochrome** | **Clone / sequence** | **Company** | **Catalog #** | **Dilution** | **Titer (ng/test)** |
| --- | --- | --- | --- | --- | --- | --- |
| **Live/dead staining (RT, 100 µL):** | | | | | | |
| L/D | Fixable Violet | - | Thermo Fisher | L34964 | 1:800 | - |
| **TM staining (37°C, 100 µL):** | | | | | | |
| MTB_30 | PE | VLPKRARLL | - | - | 1:50 | 540 |
| MTB_31 | PE | VLPAKLILM | - | - | 1:50 | 540 |
| MTB_34 | PE | LLPIKIPLI | - | - | 1:50 | 540 |
| MTB_62 | PE | ALQSAAPWL | - | - | 1:50 | 540 |
| MTB_63 | PE | ILAFEAPEL | - | - | 1:50 | 540 |
| MTB_65 | PE | ILLSRVPEL | - | - | 1:50 | 540 |
| MTB_81 | PE | VLPLAAPWL | - | - | 1:50 | 540 |
| MTB_87 | PE | KLSTLTPYL | - | - | 1:50 | 540 |
| MTB_93 | PE | RLEAVVMLL | - | - | 1:50 | 540 |
| p34 | PE | VMTTVLATL | - | - | 1:50 | 540 |
| p62 | PE | RMPPLGHEL | - | - | 1:50 | 540 |
| p44 | PE | RLPAKAPLL | - | - | 1:50 | 540 |
| **Surface staining (4°C, 100 µL):** | | | | | | |
| CD3 | BV510 | UCHT1 | Biolegend | 300448 | 1:50 | 200 |
| CD4 | PerCP-Cy5.5 | RPA-T4 | BD Biosciences | 560650 | 1:50 | - |
| CD8a | AlexaFluor700 | RPA-T8 | Biolegend | 301028 | 1:50 | 1000 |
| CD14 | APC-Cy7 | M5E2 | Biolegend | 301820 | 1:50 | 800 |
| CD16 | PE-Cy5 | 3G8 | Biolegend | 302010 | 1:100 | 60 |
| CD19 | BV570 | HIB19 | Biolegend | 302236 | 1:50 | 100 |
| CD56 | PE-CF594 | B159 | BD Biosciences | 562289 | 1:50 | - |

**Supplementary Table 5. Flow cytometry panel for extensive characterization of the total and HLA-E/*Mtb* specific immune repertoire in individuals with TBI, TBI and HIV or aTB and HIV.**

| **Marker** | **Fluorochrome** | **Clone / sequence** | **Company** | **Catalog #** | **Dilution** | **Titer (ng/test)** |
| --- | --- | --- | --- | --- | --- | --- |
| **Live/dead staining (RT, 100 µL):** | | | | | | |
| L/D | Fixable Blue | - | Thermo Fisher | L23105 | 1:1000 | - |
| **Chemokine receptor staining (37°C, 100 µL):** | | | | | | |
| CCR4 | PE-Cy7 | L291H4 | Biolegend | 359410 | 1:200 | 100 |
| CCR6 | BV605 | G034E3 | Biolegend | 353420 | 1:50 | 100 |
| CCR7 | BV421 | G043H7 | Biolegend | 353208 | 1:50 | 140 |
| CXCR3 | BUV661 | 1C6/CXCR3 | BD Biosciences | 741649 | 1:50 | 400 |
| CCR2 | BV785 | K036C2 | Biolegend | 357234 | 1:200 | 25 |
| **TM staining (37°C):** | | | | | | |
| ***Mtb* pool 1 (in 100 µL):** | | | | | | |
| MTB_31 | PE | VLPAKLILM | - | - | 1:50 | 540 |
| MTB_93 | PE | RLEAVVMLL | - | - | 1:50 | 540 |
| ***Mtb* pool 2 (in 100 µL):** | | | | | | |
| MTB_34 | PE | LLPIKIPLI | - | - | 1:50 | 540 |
| MTB_63 | PE | ILAFEAPEL | - | - | 1:50 | 540 |
| **p44 control (in 50 µL):** | | | | | | |
| p44 | PE | RLPAKAPLL | - | - | 1:50 | 270 |
| **Surface staining (4°C, 100 µL):** | | | | | | |
| CD3 | BV510 | UCHT1 | Biolegend | 300448 | 1:50 | 200 |
| CD4 | CF568 | EDU-2 | Biotium | BNC680345-500 | 1:400 | 25 |
| CD8 | BUV805 | G42-8 | BD Biosciences | 749032 | 1:400 | 50 |
| TCRαβ | AlexaFluor488 | IP26 | Biolegend | 306712 | 1:200 | 200 |
| TCRγδ | BV650 | 11F2 | BD Biosciences | 745359 | 1:50 | 400 |
| CD45RA | BUV395 | 5H9 | BD Biosciences | 740315 | 1:400 | 50 |
| CD27 | Pacific Blue | O323 | Biolegend | 302822 | 1:200 | 250 |
| CD28 | BUV563 | L293 | BD Biosciences | 748476 | 1:50 | 400 |
| CD95 | PE-Cy5 | DX2 | Biolegend | 305610 | 1:400 | 50 |
| CD62L | APC-Fire750 | DREG-56 | Biolegend | 304846 | 1:100 | 200 |
| CD25 | PE-Fire640 | M-A251 | Biolegend | 356148 | 1:50 | 400 |
| NKG2a | BUV737 | 131411 | BD Biosciences | 749682 | 1:25 | 800 |
| NKG2c | BUV615 | 134591 | BD Biosciences | 751059 | 1:100 | 200 |
| CD56 | BV750 | 5.1H11 | Biolegend | 362556 | 1:100 | 100 |
| CD16 | BUV496 | B73.1 | BD Biosciences | 741207 | 1:50 | 400 |
| CD14 | SparkBlue550 | 63D3 | Biolegend | 367148 | 1:400 | 100 |
| CD85d | PE-Dazzle594 | 42D1 | Biolegend | 338710 | 1:100 | 100 |
| CD85j | AlexaFluor647 | GHI/75 | Biolegend | 333709 | 1:25 | 1600 |
| HLA-DR | PE-Fire810 | L243 | Biolegend | 307683 | 1:100 | 50 |
| CD38 | APC-Fire810 | HIT2 | Biolegend | 303550 | 1:100 | 150 |
| CD19 | SparkNIR685 | HIB19 | Biolegend | 302270 | 1:100 | 100 |
| CD20 | PacificOrange | HI47 | LifeTechnologies | MHCD2030 | 1:25 | - |
|  | BV570 | 2H7 | Biolegend | 302332 | 1:50 | 200 |
| IgD | BV480 | IA6-2 | BD Biosciences | 566187 | 1:100 | 200 |

**Supplementary Table 6. Flow cytometry panel to determine exhaustion marker expression of KLRG1, PD-1 and 2B4 on HLA-E T cells in individuals with TBI or TBI and HIV.**

| **Marker** | **Fluorochrome** | **Clone / sequence** | **Company** | **Catalog #** | **Dilution** | **Titer (ng/test)** |
| --- | --- | --- | --- | --- | --- | --- |
| **Live/dead staining (RT, 100 µL):** | | | | | | |
| L/D | Fixable Blue | - | Thermo Fisher | L23105 | 1:1000 | - |
| **TM staining (37°C):** | | | | | | |
| ***Mtb* pool 1 (in 100 µL):** | | | | | | |
| MTB_31 | PE + APC | VLPAKLILM | - | - | 1:50 | 540 |
| MTB_93 | PE + APC | RLEAVVMLL | - | - | 1:50 | 540 |
| ***Mtb* pool 2 (in 100 µL):** | | | | | | |
| MTB_34 | PE + APC | LLPIKIPLI | - | - | 1:50 | 540 |
| MTB_63 | PE + APC | ILAFEAPEL | - | - | 1:50 | 540 |
| **p44 control (in 50 µL):** | | | | | | |
| p44 | PE + APC | RLPAKAPLL | - | - | 1:50 | 270 |
| **pCMV control (in 50 µL):** | | | | | | |
| pCMV | PE + APC | VLAPRTLLL | - | - | 1:50 | 270 |
| **Surface staining (4°C, 100 µL):** | | | | | | |
| CD14 | BV480 | M5E2 | BD Biosciences | 746304 | 1:200 | 50 |
| CD19 | CF480 | HIB19 | BD Biosciences | 568214 | 1:200 | 100 |
| CD3 | BV510 | UCHT1 | Biolegend | 300448 | 1:50 | 200 |
| CD4 | CF568 | EDU-2 | Biotium | BNC680345-500 | 1:400 | 25 |
| CD8 | BUV805 | G42-8 | BD Biosciences | 749032 | 1:400 | 50 |
| CD45RA | BUV395 | 5H9 | BD Biosciences | 740315 | 1:400 | 50 |
| CD27 | Pacific Blue | O323 | Biolegend | 302822 | 1:200 | 250 |
| CD28 | BUV563 | L293 | BD Biosciences | 748476 | 1:50 | 400 |
| NKG2a | BUV737 | 131411 | BD Biosciences | 749682 | 1:25 | 800 |
| NKG2c | BUV615 | 134591 | BD Biosciences | 751059 | 1:100 | 200 |
| CD56 | BV750 | 5.1H11 | Biolegend | 362556 | 1:100 | 100 |
| CD57 | BV421 | NK-1 | BD Biosciences | 568894 | 1:400 | 82,5 |
| CD7 | BV605 | M-T701 | BD Biosciences | 740392 | 1:100 | 200 |
| CD127 | SparkNIR685 | A019D5 | Biolegend | 351362 | 1:25 | 400 |
| ICOS | BV650 | DX29 | BD Biosciences | 563832 | 1:50 | 200 |
| 2B4 | FITC | C1.7 | Biolegend | 329506 | 1:400 | 50 |
| PD-1 | PE-Cy7 | EH12.2H7 | Biolegend | 329918 | 1:100 | 200 |
| KLRG1 | APC-Fire810 | SA231A2 | Biolegend | 367732 | 1:100 | 50 |
